# Development of a WHO Anti-HTLV Reference Panel

**DOI:** 10.1186/1742-4690-11-S1-P145

**Published:** 2014-01-07

**Authors:** Clare Morris, Elliot P Cowan

**Affiliations:** 1 Division of Virology, NIBSC, Blanche Lane, Potters Bar, Hertfordshire, UK; 2 Division of Emerging and Transfusion Transmitted Diseases, Center for Biologics Evaluation and Research, US FDA, Rockville, Maryland, USA

## 

Antibody screening tests are critical tools to identify individuals infected with HTLV-1 and HTLV-2. However, the lack of reference panels hinders the ability to evaluate existing tests and new technologies that aid in the screening for these agents. For example, some HTLV-2 subtypes may escape detection by currently available technology. The World Health Organization, through the support of the WHO Expert Committee on Biological Standardization, is in the process of generating such a reference panel.

This reference panel will best function as a tool for test assessment only if it consists of specimens representing the diversity of HTLV-1 and HTLV-2, especially those subtypes known to present challenges to detection using serological tests.

There have been challenges sourcing suitable material of sufficient titre and volume. Initial screening tests carried out by CBER/FDA and NIBSC, using a range of EIA’s, have shown a variation in titre of donated material. Two candidates, HTLV-1a and HTLV-2, have been identified and are in the process of being formulated into lyophilised preparations. Such preparations will shortly be distributed to participating laboratories for testing in a small international collaborative study. It is envisaged that additional panel members will be added as and when appropriate material is sourced.

This first WHO HTLV antibody reference panel is expected to be used by blood screening and diagnostic test developers, blood banks, hospitals and other establishments performing HTLV serology testing.

